# Modeling Tumor-Associated Edema in Gliomas during Anti-Angiogenic Therapy and Its Impact on Imageable Tumor

**DOI:** 10.3389/fonc.2013.00066

**Published:** 2013-04-04

**Authors:** Andrea Hawkins-Daarud, Russell C. Rockne, Alexander R. A. Anderson, Kristin R. Swanson

**Affiliations:** ^1^Department of Neurological Surgery, Northwestern UniversityChicago, IL, USA; ^2^Integrated Mathematical Oncology, Moffitt Cancer CenterTampa, FL, USA

**Keywords:** glioma, edema, mathematical model, anti-angiogenic therapy

## Abstract

Glioblastoma, the most aggressive form of primary brain tumor, is predominantly assessed with gadolinium-enhanced T1-weighted (T1Gd) and T2-weighted magnetic resonance imaging (MRI). Pixel intensity enhancement on the T1Gd image is understood to correspond to the gadolinium contrast agent leaking from the tumor-induced neovasculature, while hyperintensity on the T2/FLAIR images corresponds with edema and infiltrated tumor cells. None of these modalities directly show tumor cells; rather, they capture abnormalities in the microenvironment caused by the presence of tumor cells. Thus, assessing disease response after treatments impacting the microenvironment remains challenging through the obscuring lens of MR imaging. Anti-angiogenic therapies have been used in the treatment of gliomas with spurious results ranging from no apparent response to significant imaging improvement with the potential for extremely diffuse patterns of tumor recurrence on imaging and autopsy. Anti-angiogenic treatment normalizes the vasculature, effectively decreasing vessel permeability and thus reducing tumor-induced edema, drastically altering T2-weighted MRI. We extend a previously developed mathematical model of glioma growth to explicitly incorporate edema formation allowing us to directly characterize and potentially predict the effects of anti-angiogenics on imageable tumor growth. A comparison of simulated glioma growth and imaging enhancement with and without bevacizumab supports the current understanding that anti-angiogenic treatment can serve as a surrogate for steroids and the clinically driven hypothesis that anti-angiogenic treatment may not have any significant effect on the growth dynamics of the overall tumor cell populations. However, the simulations do illustrate a potentially large impact on the level of edematous extracellular fluid, and thus on what would be imageable on T2/FLAIR MR. Additionally, by evaluating virtual tumors with varying growth kinetics, we see tumors with lower proliferation rates will have the most reduction in swelling from such treatments.

## Introduction

**Figure 1:**
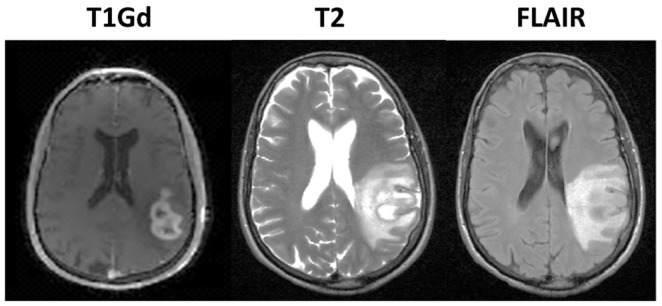
**Illustration of primary imaging modalities**. The T1Gd image will show enhancement where the contrast agent has been able to diffuse into the extracellular space where the blood brain barrier has been compromised due to tumor-induced neo-angiogenesis. The T2-weighted and FLAIR images are associated with edema or swelling; FLAIR is different from T2 in that the signal from the cerebral spinal fluid (CSF) is removed. In the case of GBM, the non-CSF T2/FLAIR enhancement is primarily vasogenic edema, defined as fluid originating from blood vessels that accumulates around cells (Marmarou, 2007). The fluid leaves the vessels due to pressure and osmotic gradients induced by the breakdown in the blood brain barrier.

Glioblastoma Multiforme (GBM) is a highly aggressive and invasive primary brain tumor. The standard treatment protocol is to surgically remove as much of the tumor as is reasonably safe, followed by a combination of chemotherapy with radiation. Despite aggressive treatment, the prognosis remains poor with a median survival time of 14 months (Stupp et al., 2005). The inability to accurately determine the extent of diffuse tumor cell infiltration of the normal brain affects the ability to assess response to treatment through clinical imaging, confounding clinical progress. Currently, clinicians rely primarily on three magnetic resonance imaging (MRI) modalities to monitor the development of the tumor, the T2 weighting, FLAIR, and T1 weighting with gadolinium contrast enhancement (T1Gd) sequences illustrated in Figure 1. However, it is known that none of these sequences are able to show the entire extent of the malignant cells (Silbergeld and Chicoine, 1997), since the abnormal regions highlighted in the MR images are as dependent on the microenvironment around the disease, particularly the vasculature, as on the tumor cells themselves.

Increased vasculature is a primary hallmark of GBM, and while angiogenesis is a hallmark of cancer in general, there are some important factors that separate GBMs from other tumors. First, the glioma cells inhabit an organ that is highly vascularized in its native state. Second, glioma cells are diffusely invasive and are known to co-opt the existing vasculature and migrate and grow along the vessels (Holash et al., 1999; Leenders et al., 2002). Nevertheless, GBMs can form hypoxic regions, often leading to regions of necrosis, and thus, downstream of this hypoxic signaling, emit an abnormally large amount of angiogenic factors such as vascular endothelial growth factor (VEGF) for the recruitment of additional vasculature, analogous to observations in solid tumors (Kerbel, 2000). This process results in the vasculature developing abnormally large vessel radial sizes and, unique to the brain, results in a breakdown of the blood brain barrier in the tumor region.

### Features characterizing MR imaging observation

In the case of GBM, the enhancing abnormalities on all of the primary MR imaging modalities, T1Gd, T2, and FLAIR primarily result from a compromised blood brain barrier. The T1Gd image signal is enhanced where the contrast agent has been able to leak into the extracellular space through breakdowns in the blood brain barrier due to tumor-induced neo-angiogenesis. The T2-weighted and FLAIR images show edema or swelling; FLAIR is different from T2 in that the signal from the cerebral spinal fluid (CSF) is inverted. In the case of GBM, the non-CSF T2/FLAIR hyperintense signal is primarily vasogenic edema, defined as fluid originating from blood vessels that accumulates around cells (Marmarou, 2007). The fluid leaves the vessels due to pressure and osmotic gradients induced by the breakdown in the blood brain barrier. Thus, whilst changes in any primary imaging modality (T1Gd or T2/FLAIR) are often interpreted as corresponding to changes in tumor cell density, they may be artifacts of MR imaging.

### The role of anti-angiogenics in GBM

The concept of anti-angiogenic treatment for cancer has been popular ever since the landmark paper by Folkman (1971) stating that malignant tumors were angiogenesis-dependent and has been used with some success for other solid tumors in combination with chemotherapy (Hurwitz et al., 2004; Sandler et al., 2006). Since a defining hallmark of GBM is increased vasculature through endothelial cell proliferation (Louis et al., 2007), this disease seems like an obvious candidate for vascular targeting treatment. However, the differences between the vasculature in GBMs and other solid tumors produce different treatment effects. In GBMs, one of the effects of anti-angiogenic treatments is to, at least transiently, repair the blood brain barrier and allow the vessels to return to their normal radial size, increasing their efficiency (Jain, 2005; Batchelor et al., 2007) – referred to as vascular normalization. Ostensibly, this improved efficiency of the vasculature is not the desired impact, though it may help in delivery of other therapeutic agents.

**Figure 2:**
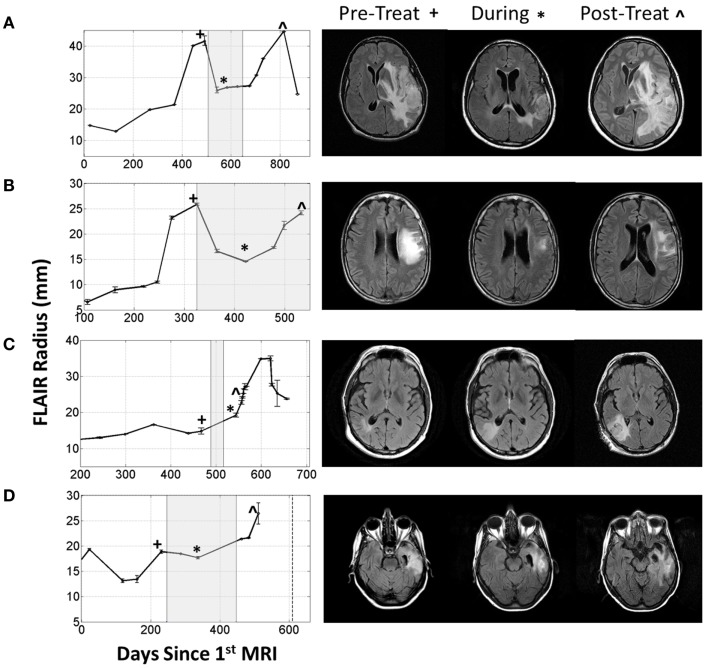
**Four patients with varying imageable responses to anti-angiogenic treatment**. Treatment period indicated with gray box on radius plots. **(A)** Patient 1: A 48-year-old male with Grade III glioma is seen to have significant reduction of enhancing lesion during treatment but recurs almost immediately after treatment is stopped, **(B)** Patient 2: a 55-year-old male with GBM initially responds to treatment but even while being treated the enhancing region is seen to enlarge again, **(C)** Patient 3: a 61-year-old male with GBM seems to have no response to treatment and the enhancing region seems to grow faster after treatment, and **(D)** Patient 4: a 66-year-old female with GBM has imaging stabilized during treatment, but enhancing region begins growing again once treatment is stopped.

More concerning, however, is that this normalization may directly impact the efficacy of the MR imaging. It is possible for glioma patients with enhancing lesions on T1Gd and T2/FLAIR imaging to have decreased enhancement within a day of anti-angiogenic treatment (Batchelor et al., 2007; Norden et al., 2008), as illustrated by patients 1 and 2 in Figure 2, but upon stopping treatment, the imageable lesion is even larger and more disperse than before (Iwamoto et al., 2009). However, responses are varied; a patient may see no deflection in growth but faster growth after treatment, such as the third patient in Figure 2, or see stabilized disease returning to the previous growth patterns after treatment, as illustrated by the fourth patient in Figure 2. These patients were consented to this study with approval by the local institutional review board at either the University of Washington or the University of California, Los Angeles, and their relevant demographic and therapeutic information is given in Table 1. These conundrums have led to two hypotheses: first, anti-angiogenic treatment has minimal cytotoxic effect but does influence the imaging so that the tumor cannot be effectively visualized, and second, that the treatment may be selecting for a more aggressively invasive phenotype (Verhoeff et al., 2009; Keunen et al., 2011).

**Table 1:** **Demographic and treatment information corresponding to patients in Figure 2**.

	Age	Sex	Grade	Race	XRT dose (cGy)	Concurrent TMZ	Bev given at recurrence	Concurrent therapies with Bev
Patient 1	48	M	III	Caucasian	Given, but unknown dosage	Y	Y	Irinotecan, dexamethasone
Patient 2	55	M	IV	Caucasian	6000	Y	Y	Carboplatin
Patient 3	61	M	IV	Unknown	6000	Y	Y	Irinotecan
Patient 4	66	F	IV	Caucasian	6120	Y	Y	Irinotecan

Previous studies have shown the use of anti-angiogenic drugs, specifically bevacizumab (Avastin), tends to increase progression-free survival and reduce symptoms of recurrent GBMs, but they have failed to consistently show a significant increase in overall survival and there is concern that the measured radiographic responses do not reflect changes in tumor cell counts (Verhoeff et al., 2009; Deming, 2012). Additionally, animal studies have revealed that treatment with anti-angiogenic drugs may be creating an environment favorable for local invasion and metastasis (Ebos et al., 2009; Pàez-Ribes et al., 2009). Even though there is a lack of significant evidence for increased overall survival after bevacizumab, and it is possible that treatment selects for a more invasive phenotype, the increase in quality of life for some of the patients, due to the relief from edema-related symptoms, means anti-angiogenic therapy is an attractive and relevant treatment option. However, the inability to determine *a priori* which patients will receive more benefit than harm from anti-angiogenic therapy ultimately keeps clinicians wary (Deming, 2012).

In this paper, we aim to illustrate how a previous mathematical model of glioma growth can be extended to explicitly incorporate edema formation allowing us to directly characterize and potentially predict the effects of anti-angiogenics on imageable tumor growth. The ultimate goal of this model is to help the treatment planning process by identifying exactly those patients that would receive the most benefit from anti-angiogenic treatment.

## Materials and Methods

### The proliferation-invasion-hypoxia-necrosis-angiogenesis-edema model

Over the last decade we have made a significant effort toward the development of patient specific mathematical models of GBM that are able to capture the growth kinetics of individual patients (Swanson, 1999; Swanson et al., 2000, 2002a,b; Harpold et al., 2007; Rockne et al., 2010). The simplest form of the model, referred to as the Proliferation-Invasion (PI) model is based on patient specific net rates of proliferation and invasion and has been successful in predicting untreated growth rates for individual patients (Harpold et al., 2007) and providing predictions of outcomes following surgical resections (Swanson et al., 2003), chemotherapy (Swanson et al., 2002a,b, 2003), and radiation (Rockne et al., 2010), while also providing insight into glioma ontogeny (Bohman et al., 2010).

### A mathematical model of the angiogenic cascade in glioblastoma

The Proliferation-Invasion-Hypoxia-Necrosis-Angiogenesis (PIHNA) model first discussed in Swanson et al. (2011) incorporates the angiogenic cascade and characterizes malignant gliomas with relative proportions of well-oxygenated “normoxic” tumor cells, (*c*), poorly oxygenated hypoxic tumor cells, (*h*), necrotic cells, (*n*), and vascular, or endothelial cells, (*v*), along with a generic population of angiogenic factors, (*a*) (Swanson et al., 2011). In words, it assumes the level of nutrients present in the local microenvironment, as inferred from the number of vasculature cells, determines whether the present tumor cells will exhibit normoxic or hypoxic phenotypes. That is, if there is a sufficient level of nutrients present, the cells will remain normoxic, but if the nutrient level falls below a given threshold, the cells will become hypoxic. If the nutrients provided by the vasculature fall below an even lower threshold value, the hypoxic cells will undergo necrosis, at a rate of α*_h_* (1/year) and remain in the necrotic cell population. Normoxic tumor cells are allowed to move (invade) and divide while, due to restricted amounts of nutrients, the hypoxic cells are only allowed to move. The hypoxic cells produce a large amount of angiogenic factors which ultimately cause an increase in the number of vasculature cells. The system is described with a mathematical model composed of the following five coupled reaction-diffusion equations:

(1) $$\begin{array}{l} \frac{\partial c}{\partial t}=\overset{\begin{array}{c} \text{Net dispersal of normoxic}\\ \text{glioma cells} \end{array}}{\overset{︷}{\nabla \cdot \text{(}D(x)\;(1-T)\;\nabla c)}}+\overset{\begin{array}{c} \text{Net proliferation of normoxic}\\ \text{glioma cells} \end{array}}{\overset{︷}{\rho \;c\;(1-T)}}\\ \;+\overset{\begin{array}{c} \text{Conversion of hypoxic}\\ \text{to normoxic} \end{array}}{\overset{︷}{\gamma \;h\;V}}-\overset{\begin{array}{c} \text{Conversion of normoxic}\\ \text{to hypoxic} \end{array}}{\overset{︷}{\beta \;c\;(1-V)}}\\ \;-\overset{\begin{array}{c} \text{Conversion of normoxic}\\ \text{to necrotic} \end{array}}{\overset{︷}{\alpha_{n}\;n\;c}}\\ \frac{\partial h}{\partial t}=\overset{\begin{array}{c} \text{Dispersal of hypoxic}\\ \text{glioma cells} \end{array}}{\overset{︷}{\nabla \cdot (D(x)\;(1-T)\;\nabla h)}}-\overset{\begin{array}{c} \text{Conversion of hypoxic}\\ \text{to normoxic} \end{array}}{\overset{︷}{\gamma \;h\;V}}\\ \;+\overset{\begin{array}{c} \text{Conversion of normoxic}\\ \text{to hypoxic} \end{array}}{\overset{︷}{\beta \;c\;(1-V)}}-\overset{\begin{array}{c} \text{Conversion of hypoxic}\\ \text{to necrotic} \end{array}}{\overset{︷}{(\alpha_{h}h\;(1-T)+\alpha_{n}\;nh}}\\ \frac{\partial n}{\partial t}=\overset{\text{Conversion of hypoxic, normoxic, and vasculature to necrotic}}{\overset{︷}{\alpha_{h}h\;(1-V)+\alpha_{n}\;n\;(c+h+\nu )}}\\ \frac{\partial \nu }{\partial t}=\overset{\begin{array}{c} \text{Dispersal of}\\ \text{vasculature} \end{array}}{\overset{︷}{\nabla \cdot (D_{\nu }(x)\;(1-T)\;\nabla \;\nu )}}+\overset{\begin{array}{c} \text{Net proliferation}\\ \text{of vasculature} \end{array}}{\overset{︷}{\mu \frac{a}{K_{m}+a}\;\nu \;(1-T)}}\\ \;-\overset{\begin{array}{c} \text{Conversion of vasculature}\\ \text{to necrotic} \end{array}}{\overset{︷}{\alpha_{n}\;n\;\nu }}\\ \frac{\partial a}{\partial t}=\overset{\begin{array}{c} \text{Net dispersal}\\ \text{of angiogenic factors} \end{array}}{\overset{︷}{\nabla \cdot (D_{a}\;\nabla a)}}+\overset{\begin{array}{c} \text{Net production of}\\ \text{angiogenic factors} \end{array}}{\overset{︷}{\delta_{c}\;c+\delta_{h}\;h}}\\ \;-\overset{\begin{array}{c} \text{Net consumption of}\\ \text{angiogenic factors} \end{array}}{\overset{︷}{q\mu \frac{a}{K_{m}+a}\nu (1-T)-\omega a\nu }}-\overset{\begin{array}{c} \text{Decay of}\\ \text{angiogenic factors} \end{array}}{\overset{︷}{\lambda a}}. \end{array}\}$$

In these equations, *D*(*x*) is the net rate of invasion (mm^2^/year). Glioma cells migrate faster along myelinated axons in the white matter than in the dense and less structured cortical gray matter. For this reason, we consider the net rate of invasion as piecewise constant, with non-zero values in the gray and white matter, *D*_g_ and *D*_w_, respectively, with *D*_w_ > *D*_g_, and zero in the regions of cerebral spinal fluid. Additionally, ρ (1/year) is the net proliferation rate of the normoxic cells, γ (1/year) and β (1/year) are the maximum conversion rates between the hypoxic and normoxic cell populations, α*_n_* (1/year) is the rate at which cells undergo necrosis when in contact with necrotic cells (contact necrosis), α*_h_* (1/year) is the rate of conversion of hypoxic cells to necrotic cells when nutrient levels fall too low, *D_v_* (mm^2^/year) is the rate of dispersal of vasculature cells, estimated from Sherratt and Murray (1990), Levine et al. (2001) μ (1/year) is the vasculature proliferation rate, estimated from Xiu et al. (2006), *T* = (*c* + *h* + *n* + *v*)/*K* (dimensionless), where *K* is the carrying capacity (cells/mm^3^), and *V* = *v*/(*v* + *c* + *h*) (dimensionless) and is a surrogate for the local vasculature efficiency. Angiogenic factors are produced by both normoxic and hypoxic cells with rates δ*_c_* (1/year) and δ*_h_* (1/year) respectively, with δ*_h_* > δ*_c_* and are consumed by the vasculature for both regular vasculature maintenance [with rate ω (1/year)] and for vasculature proliferation [with rate *q* (1/year)]. Finally, the angiogenic factors are assumed to decay over time with rate λ (1/year) and disperse with rate *D_v_* (mm^2^/year). Values for parameters related to the angiogenic factors were derived in part from work done in Levine et al. (2001), Serini et al. (2003), Mac Gabhann and Popel (2004). The reader is referred to (Swanson et al., 2011) for further details.

It is known that GBM tumors are extremely genetically heterogeneous both within a single tumor and between different tumors (Dunn et al., 2012). A large effort has been put forth to identify subtypes of GBMs by their dominating genotype (Verhaak et al., 2010). While the model parameters do not directly try to capture effects of single mutations, it is our belief that the dominating genotypes characterizing subtypes of GBMs ultimately result in different net rates of proliferation and invasion which would be used in our model. For example, pro-neural tumors are more likely to have the IDH-1 mutation and be secondary GBMs. In our model, this would manifest as a low-D, low-ρ tumor which begins as low grade and progresses into higher grade/malignancy.

In this model, there are different cell populations, normoxic, hypoxic, and necrotic competing for space and each with differing phenotypes: normoxic cells proliferating and invading, hypoxic cells only invading, and necrotic cells which are dead and just taking up space. Since each cell population is evolving in space and time, there is an effective spatial heterogeneity of predicted proliferative activity across space and time which could be analogized to heterogeneous Ki67 labeling across glioma specimens. Thus, while this model is attempting to capture the overarching phenotype of different tumors and assumes global constants for individual tumors, spatial heterogeneity in behavior is possible due to regional levels of vasculature which may result in hypoxia and/or necrosis. We also remark the aim of this model is not to predict cell-level behaviors (ex. Ki67), rather, use information obtained from routine imaging to quantify and explain imaging scale behavior and evolution.

**Figure 3:**
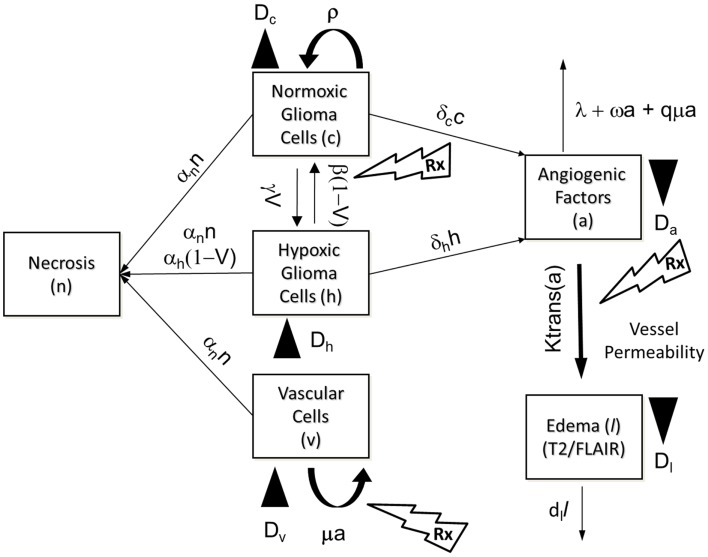
**Schematic of the PIHNA-E model**. The main components of the model are seen in the flow chart: *c* represents the normoxic glioma cells, *h* the hypoxic gliomas cells, *v* the vascular endothelial cells, *n* the necrotic cells, *a*, the angiogenic factors, and *l* the edematous fluid. Depending on the level of oxygen, normoxic, and hypoxic cells will undergo phenotypic switching. If oxygen levels fall too far and are not compensated for by sufficient angiogenesis, the hypoxic cells will undergo necrosis. Additionally, all cells will undergo necrosis if in contact with necrotic cells. Both hypoxic and normoxic cells release angiogenic factors into the extracellular space which recruit additional vasculature to increase the levels of oxygen. The angiogenic factors are removed from the system by interaction with vascular cells or natural decay. The local levels of angiogenic factors are indicative of the local degree of vessel permeability. Edematous liquid exits the vasculature where the permeability, *K*_trans_(*a*), allows and enters the extracellular space where it diffuses and will be removed at rate *d_l_*.

The PIHNA model only captures cellular species and angiogenic factors. While these all have an impact on what is ultimately seen on MR imaging, in and of themselves they are not sufficient to describe enhancing regions of T2 and T1Gd MR images. Here we extend the PIHNA model to capture the imaging responses post anti-angiogenic treatment. To achieve this, we add to the model one additional element: edema, (*l*), to create the merged proliferation-invasion-hypoxia-necrosis-angiogenesis-edema (PIHNA-E) model. A schematic of the six species interactions is shown in Figure 3.

### A mathematical model for tumor-induced edema formation

Generically, edema refers to a swelling phenomenon. While there are different types of cerebral edema, in the case of GBM, it is almost exclusively vasogenic edema which results from fluid and protein leakage from the breakdown of the blood brain barrier (Marmarou, 2007). Over the last few decades, there have been quite a few attempts to model vasogenic edema (Rapoport, 1978; Kumagai, 1986; Nagashima et al., 1990) and its associated interstitial pressure and interstitial fluid velocity (Baxter and Jain, 1989, 1990). These models were primarily based on Starling’s equation which describes fluid exchange between compartments due to pressure and osmotic gradients. These models are very detailed and are generally solved on shorter time scales, i.e., a few days versus months. In our efforts, while we do have interest in the specific mechanisms, we will take a coarser grained approach allowing us to approximate the phenomena over longer time scales relevant to tumor growth kinetics.

To begin, we make the simplifying assumption that the edema is only composed of fluid which has leaked into the extra-cellular space and has not yet been reabsorbed into the system. This fluid is assumed to leak into the extracellular space where the blood brain barrier has been compromised. From the PIHNA model, we can approximate these regions along with the degree of permeability from the local levels of present angiogenic factors. Once the fluid is in the extra-cellular space it moves via diffusion and is reabsorbed into the system at a constant rate. This process is written in the form of a partial differential equation as:

(2) $$\overset{\text{Change}\;\text{in}\;\text{time}\;\text{of}\;\text{fluid}}{\overset{︷}{\frac{\partial l}{\partial t}}}=\overset{\text{Fluid diffusion}}{\overset{︷}{\nabla \cdot \left(D_{l}\nabla l\right)}}+\overset{\text{Leakage}}{\overset{︷}{K_{\text{trans}}\left(a\right)\cdot \left(l_{v}-l\right)}}-\overset{\text{Drainage}}{\overset{︷}{\delta_{l}l}}.$$

Here *l* is the concentration of edematous fluid, *D_l_* (mm^2^/year) is the diffusion rate of the edematous liquid which would be analogous to an Apparent Diffusion Coefficient, ADC, value derived from diffusion-weighted MRI (Moritani, 2009), *l_v_* (fluid/mm^3^) is the normal level of fluid in the vasculature, δ*_l_* (1/year) is the reabsorption rate, and *K*_trans_ (1/year) is the transmission rate [analogous to the value *K*_trans_ measured on dynamic contrast enhanced MRI (DCE-MRI)], capturing the permeability surface area product per unit volume of tissue (Tofts, 1991) and is assumed to depend on the level of angiogenic factors, *a*, present. Homogeneous Neumann boundary conditions are assumed at the boundary of the brain to ensure no fluid leaves the brain.

The dependence of the *K*_trans_ coefficient on the angiogenic factors is assumed to take a Michaelis–Menten type I form:

(3) $$K_{\text{trans}}\left(a\right)=K_{\text{max}}\frac{a}{a+K_{\text{half}}}$$

to reflect that VEGF (also known as vascular permeability factor, VPF) strongly influences vascular permeability (Bates, 2010). Here *K*_max_ (1/year) is the maximum possible value of *K*_trans_, the value of which is calibrated to Grade IV gliomas (Patankar et al., 2005), and *K*_half_ (angiogenic factors/mm^3^) is the concentration of *a* at which *K*_trans_ reaches half of its maximum value. Thus, as the concentration of the angiogenic factors increases, the degree of vessel permeability will also increase until saturated.

### Modeling anti-angiogenic treatment

The PIHNA-E model describes the evolution of the tumor and its microenvironment in an untreated context. By understanding the premise of how specific therapies are meant to alter the system, one can also model the effects of various treatments. Here we are interested in anti-angiogenic treatment and, while there are many different types of drugs for this action, will focus on the drug bevacizumab.

Bevacizumab is a drug specifically targeted at the molecule vascular endothelial growth factor A (VEGF A). This particular angiogenic factor stimulates the growth of new vessels by binding with the vascular endothelial growth factor receptor (VEGFR2) on endothelial cells. Bevacizumab inhibits angiogenesis by binding to the free molecules of VEGF A and preventing them from binding to VEGFR2. An unintended consequence of this drug, however, is that beyond preventing the growth of new vessels, it also “normalizes” pre-existing vasculature (Jain, 2005; Verhoeff et al., 2009). That is, once the levels of stimulating angiogenic factors are reduced, the vessels are able to repair their leakiness and return to a normal size – making them more efficient nutrient deliverers. In our model, both of these phenomena can be captured by requiring higher levels of angiogenic factor to be present to have the same level of “action” in the contexts of both vessel proliferation and vessel permeability. Additionally, since the treatment is making the vessels more efficient, the level of vasculature needed for a cell to be normoxic will decrease, which we can capture by modifying the cell conversion rates from hypoxic to normoxic and from normoxic to hypoxic. Treatment is approximated by decreasing the parameter for conversion from normoxic to hypoxic (β) by a factor of 10, increasing the parameter for conversion from hypoxic and normoxic (γ) by a factor of 10, and increasing the required levels of angiogenic factors for inducing vascular growth and vessel permeability by 2 as supported by the studies in Desjardins et al. (2007), Zhang et al. (2009). The treatment modification of β and γ is representative of a dramatic increase in the efficiency of the blood vessels, though exact changes are not available from experimental data.

### Simulations of glioblastoma growth and response to anti-angiogenic therapy

For simplicity, we consider in all simulations here a two-dimensional tumor growing on one axial slice of the brain, with the brain geometry defined from the BrainWeb atlas (Cocosco et al., 1997). The brain is primarily composed of three different types of matter, CSF, gray matter, and white matter. Glioblastomas originate in gray or white matter and due to physical barriers will not enter into the regions of CSF. New mass will often deform the barriers, a phenomena called mass effect, and while there are some models that attempt to capture this (Clatz et al., 2005; Mohamed and Davatzikos, 2005; Hogea et al., 2008), here the brain is considered a stationary domain.

In all simulations, the domain is taken to be a slice of human brain embedded in a grid [0, 147] mm × [0, 185] mm and the equations are spatially discretized on a grid with resolution of 1 mm × 1 mm using first order accurate finite volumes. Time integration is done with an operator splitter technique utilizing backward Euler for the diffusion terms and the TR-BDF2 algorithm (Leveque, 2005) for the reaction terms with a time step size of 1 day. The simulations were initiated with a small amount of normoxic cells distributed as

$$c_{0}\left(x,\;y,\;t=0\right)=1000*\exp\left(-\left(100\left[{\left(x-x_{0}\right)}^{2}+{\left(y-y_{0}\right)}^{2}\right]\right)\right)$$

where (*x*_0_, *y*_0_) = (103, 83). Vasculature is set at 3% of the cell carrying capacity in all gray and white matter based on estimates from Blinkov and Glezer (1968), and all other quantities in the PIHNA-E model are initiated to zero. Unless otherwise stated, parameter values used in simulations for the edema equation are in Table 2, the additional parameter values are taken as specified in Swanson et al. (2011).

**Table 2:** **Parameter values for the edema equation used in all simulations unless stated otherwise in the text**.

Parameter	Definition	Value	Reference
*K*_half_	Michaelis–Menten half-max of response of ECs to angiogenic factors	5.75e−7 (mmol/cc tissue)	Derived from Mac Gabhann and Popel (2004)
*D_l_*	Net rate of edematous fluid diffusion	0.77e−3 (mm^2^/s)	Chosen as average ADC value in normal brain tissue as given in Moritani (2009)
*K*_max_	Maximum *K*_trans_ value in response to angiogenic factors	36 (1/day)	Taken to match the maximum *K*_trans_ value observed in Grade IV gliomas in Patankar et al. (2005)
δ*_I_*	Edematous fluid reabsorption rate	0.3 × *K*_max_ (1/day)	Assumed proportional to vessel permeability

*All other parameter values are taken as described in Swanson et al. (2011)*.

## Results

### Decoupling imaging changes from tumor response

**Figure 4:**
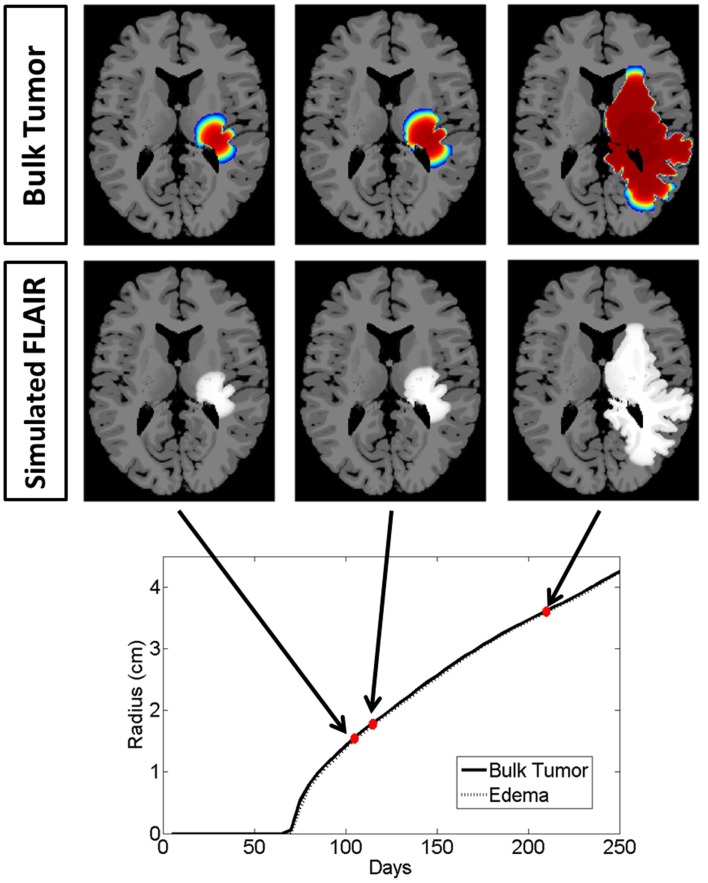
**Illustrated here is the comparison of the simulated disease burden to what would be imageable on a FLAIR MRI in an untreated context for one set of growth parameters corresponding to an aggressive GBM**. Below the plot shows the spherically symmetric equivalent radial growth of the regions containing tumor cells above a threshold and edematous fluid above a threshold. In the untreated context, these lines are nearly identical.

**Figure 5:**
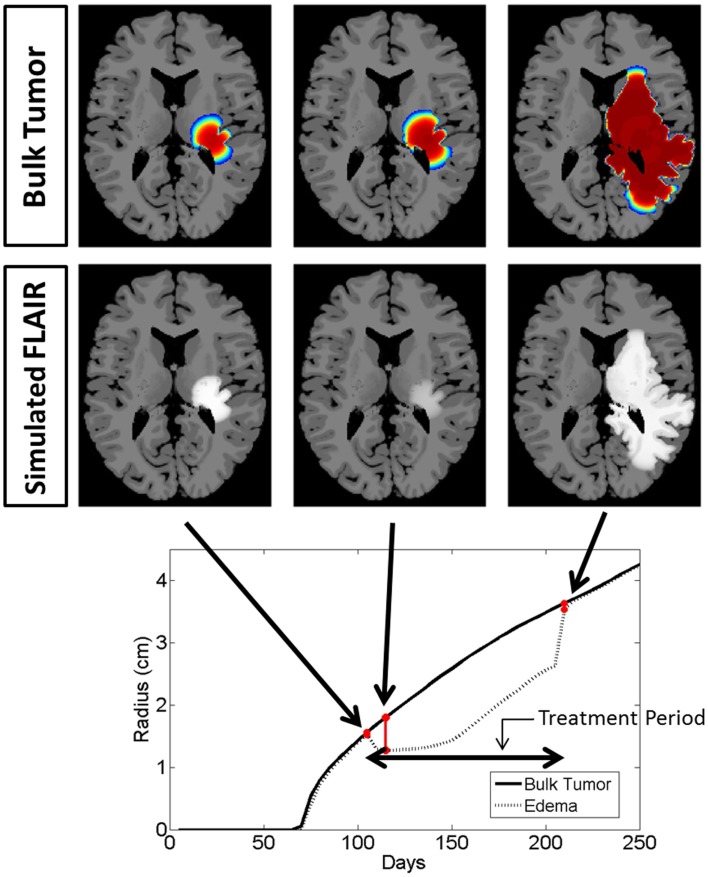
**Comparison of the simulated disease burden to what would be imageable on a FLAIR MRI in a treated context for the same set of growth parameters as shown in Figure 4**. Below the plot shows the spherically symmetric equivalent radial growth of the regions containing tumor cells above a threshold and edematous fluid above a threshold. Once treatment has begun, we see a drop in the levels of edema. Upon termination of the treatment, the edematous volume is seen to once again increase to the same size of the volume of tumorous cells.

To highlight the real impact of anti-angiogenic treatment as captured by our model, we chose parameter values that represent a patient with an aggressive GBM (net invasion rates *D*_w_ = 53 mm^2^/year and *D*_g_ = 0.53 mm^2^/year and net proliferation rate ρ = 75 1/year) and simulate tumor growth without treatment (Figure 4) and then compare to tumor growth with treatment (Figure 5). For comparison to what was done in previous work (Swanson et al., 2008a, 2011; Rockne et al., 2010), we refer to the region with total cell density summing to 80% of the cell carrying capacity (*K*) to correspond to what would enhance on the T1Gd, and started treatment when the T1Gd spherically symmetric equivalent radius was equal to 1 cm and was terminated 100 days later. This is consistent with a typical size of an abnormality seen clinically for consideration of anti-angiogenic treatment. Although treatment length can vary, 100 days is representative of the length of a typical course of treatment with anti-angiogenics in human GBM.

Snapshots of the untreated case are shown in Figure 4 with the analogous snapshots corresponding to the same time points of the treated tumor being shown in Figure 5. The top row in both Figures 4 and 5 shows the density of the bulk tumor (the summed density of all the cell populations: normoxic, hypoxic, necrotic, and vasculature). The second row shows what the simulated FLAIR corresponding to the microenvironmental levels of edematous extracellular fluid. These figures also contain radial growth plots showing the equivalent spherical radii for the regions of interest corresponding to the tumor and the edema. The line representing the bulk tumor is calculated from the volume of tissue containing abnormal cells, normoxic, hypoxic, and necrotic, at levels greater than or equal to 16% of the carrying capacity, i.e., a density five times lower than what can be visualized on T1Gd (Swanson et al., 2008b). The edema radius was defined by considering the volume containing edematous fluid above 50% of the fluid level in the capillaries. There is no literature to guide the choice of the cutoffs for fluid volume constituting T2/FL visible edema. Thus, cutoffs were chosen to roughly match clinically observed behavior.

In the untreated case (Figure 4) the radial plot shows the size of the region impacted by edema evolves very similarly to the size of the region occupied by the bulk tumor throughout the entire course of growth. In the treated case (Figure 5), the edema grows at the same rate as the bulk tumor until the treatment begins at which point the edema begins to decline. Edema begins to increase again once the angiogenic factors have been able to accumulate at levels which overcome the impact of the anti-angiogenic drug. Once treatment is terminated the edema levels rise to again occupy a region of the same size as the bulk tumor.

These simulations support the hypothesis that anti-angiogenic treatment may not have a significant effect on the growth dynamics of the overall cell populations, while having a large impact on the level of edematous extracellular fluid and thus on what would be imageable on T2/FLAIR MRI. This is also in agreement with the current understanding that anti-angiogenic treatment serves as a surrogate to steroids for reducing swelling.

### Exploring response across tumor kinetics

The virtual control experiment illustrated in Figures 4 and 5 is only providing insight into tumor/edema response for the case of one set of tumor growth kinetics. However, the range of radiographic response patterns seen clinically is broad, a few examples of which are shown in Figure 2. Previous work has shown patient specific values of net proliferation and invasion range over many orders of magnitude (Tracqui et al., 1995; Harpold et al., 2007). To investigate if the different types and extents of radiographic responses could be explained by different underlying tumor growth kinetics, we simulated tumor growth and the associated edema under treatment for many different combinations of net proliferation rates, ρ, and net invasion rates, *D*_w_ and *D*_g_.

**Figure 6:**
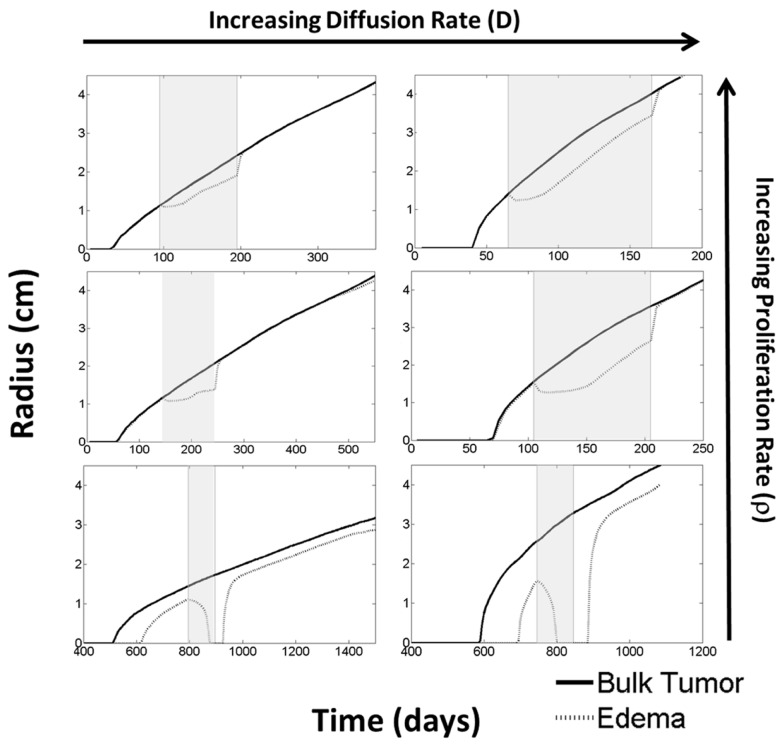
**Using the PIHNA-E model, holding all parameters constant except for *D* and ρ, one can observe many different responses to anti-angiogenic treatment in terms of the levels of edema**. These responses vary from complete disappearance of imageable edema to stabilized lower levels to lowered levels of edema that continue to increase. When treatment is terminated, however, edema levels are always seen rise to once again reflect more closely the underlying disease burden. Treatment times are indicated by the gray boxes. These simulations suggest that the majority of imaging responses can be explained by considering how the drug impacts the tumor microenvironment alone without cytotoxic affects. Additionally, they represent a possible mechanism for identifying the patients who will receive significant benefit from the treatment.

For all cases, treatment was started when the tumor reached a simulated 1 cm T1Gd radius for a total of 100 continuous days. Treatment was implemented in the same manner as the first case. Illustrative results are shown in Figure 6 from simulations with ρ = [5, 75, 125] (1/year) and *D*_w_ = [5.3, 53] (mm^2^/year) and *D*_g_ = *D*_w_/10 consistent with the observed range hypothesized in human gliomas (Harpold et al., 2007) and since observed (Wang et al., 2009; Rockne et al., 2010).

From these six scenarios, summarized in Figure 6, we were able to observe a few trends. First, none of the simulations showed a significant change in the bulk tumor growth rate after treatment had begun. However, levels of edema were impacted and by different degrees in each of the simulations. In general, tumors with higher proliferative capacities (higher ρ’s), due to their higher metabolic needs, have larger regions of hypoxia and thus produce greater levels of angiogenic factors. Treatment of these tumors initially reduces the level of edema, however, the tumor quickly produces enough angiogenic factors to continue progressing under imaging. In contrast, the slower growing tumors (low ρ) produce low levels of angiogenic factors and treatment may result in a complete disappearance of the abnormality on imaging. Additionally, higher dispersion rates (high *D*_w_ and *D*_g_) reduce the local metabolic needs and the production of angiogenic factors. Thus, the imaging of tumors with high *D*_w_ and high *D*_g_ improve for a longer time, however, ultimately the tumor does still produce enough angiogenic factors to be seen progressing on imaging. For all cases, once treatment is discontinued, the edema levels quickly rise to again reflect the underlying tumor burden.

What is particularly encouraging is that many of the different behaviors observed for the four patients illustrated in Figure 2 can be connected to different simulation predictions. For example, the first patient’s behavior is analogous to the moderate proliferation rate with a low invasion rate in that the imageable lesion initially decreased, stabilized, but after treatment dramatically increased. Additionally, the second patient can be compared to either the moderate or highly proliferative rate with a high invasion rate where the hyperintensity is seen to decrease at the beginning of treatment, but even while treatment is continuing, start growing again.

## Discussion

Anti-angiogenics remain a controversial form of treatment for GBM due to the difficulty in assessing tumor response using MR imaging. The resulting reduction in swelling and related symptoms for a subset of patients keeps it an attractive option despite the lack of evidence of an increase in overall survival and the possibility of the treatment selecting for a more aggressive phenotype (Ebos et al., 2009; Pàez-Ribes et al., 2009; Verhoeff et al., 2009; Keunen et al., 2011). It is unclear how anti-angiogenic treatment impacts the cell phenotypes present and there is not yet a deep enough understanding or a unifying theory to provide explanation for all the different response patterns. Thus, *a priori* identification of patients who will receive a significant symptom-reduction benefit remains difficult. As there are other side effects and consequences from anti-angiogenic therapy, being able to make this early distinction would help remove the controversial nature of this therapy.

The model developed in this work, built on the PIHNA model for glioma proliferation and invasion (Swanson et al., 2011; Gu et al., 2012), is meant to illustrate a first step toward the creation of a tool for identifying patients who will receive the greatest benefit from anti-angiogenic treatment. It captures the formation of edema caused by leaky vasculature, and is thus able to decouple what would be seen on the T2/FLAIR MRI from the true underlying disease burden. In effect, this would help the clinicians to “turn the light back on” by being able to infer the disease burden that lies beyond what is captured by imaging alone. While this model is not meant to capture individual cell behavior, it does provide a map between overall tumor growth kinetics and treatment response on the imaging/continuum scale.

Many simplifying assumptions have been made in the creation of this model such as ignoring the possible direct impact of anti-angiogenic therapy on cell proliferation, the likely presence of thrombosis (Tehrani et al., 2008), and that the hyperintensity on the T2/FLAIR image is entirely a result of fluid leaking from the vasculature. The model is clearly incomplete, and future modifications of the model will need to consider these other phenomena as well as other possible factors on T2/FLAIR hyperintensity, such as higher cell density and additional cytoplasm. However, even in its current state, it has been able to exhibit many of the types of response patterns observed clinically. It is particularly encouraging because the modeling effects of treatment were held constant and only varying the net dispersal and net proliferation rates, *D* and ρ, respectively, was sufficient to produce a wide range of imaging responses analogous to what is seen clinically. That is, by modeling the treatment in the exact same way for different values of proliferation and diffusion in the tumor growth model, the visible levels of edema are seen to respond in different ways. In general, the simulations predict edema (swelling) to decrease, supporting the role of these drugs as surrogates for steroids for reduction of symptoms, analogous to the most current understanding (Deming, 2012). Though, the model clearly illustrates that not all patients would receive the same benefit.

Another interesting implication from these simulations is that while different imaging responses to treatment were achieved for the same treatment conditions, in all cases the bulk tumor is seen to progress with little deflection in overall tumor growth rates. This result could be considered evidence against the cytotoxic effects of anti-angiogenic treatment when administered exclusively, also in agreement with current clinical understanding (Verhoeff et al., 2009). While this work is not directly speaking to survival outcomes, we remark that these results highlight the potential for the mathematical model paradigm to serve in evaluating clinical trial outcomes by analyzing relative benefit from anti-angiogenics, especially in the case of low-N trials. As a particular example, such models may have the potential to be applied to patient cohorts for exploring how differential effects of anti-angiogenics on imaging may or may not relate to overall outcomes.

A drawback of this model is the large number of parameters required. In this document, we assumed the primary influential factor were the net rates of invasion and proliferation and thus held all other parameters constant. These other parameters are likely different patient to patient, however, as demonstrated here, changes in the small number of parameters are sufficient to produce a wide variety of imaging responses. While future sensitivity analysis is required, we believe the work is here is evidence that a complicated explanation for the different imaging responses to anti-angiogenic therapy may not be needed. Major next steps of this work will involve developing techniques for obtaining patient specific growth parameters from pre-treatment images which we believe will be successful from previous accomplishments with a simpler model capturing just the proliferation and invasion tumor characteristics PI (Swanson, 1999, 2002; Swanson et al., 2000, 2003, 2004, 2008a; Szeto et al., 2009; Wang et al., 2009; Rockne et al., 2010).

Also, modification of the model to capture the pressure induced from the vasogenic edema and possible herniation would allow for deeper understanding of the steroid-like reduction in swelling. For each new feature eventually added validation tests will be required, however, we believe the results presented here in and of themselves represent a significant step in overcoming clinical imaging restrictions with mathematical models.

## Conflict of Interest Statement

The authors declare that the research was conducted in the absence of any commercial or financial relationships that could be construed as a potential conflict of interest.
